# Penetration Depth Measurement of Near-Infrared Hyperspectral Imaging Light for Milk Powder

**DOI:** 10.3390/s16040441

**Published:** 2016-03-25

**Authors:** Min Huang, Moon S. Kim, Kuanglin Chao, Jianwei Qin, Changyeun Mo, Carlos Esquerre, Stephen Delwiche, Qibing Zhu

**Affiliations:** 1 Key Laboratory of Advanced Process Control for Light Industry, Ministry of Education, Jiangnan University, Wuxi 214122, China; huangmzqb@163.com (M.H.); zhuqib@163.com (Q.Z.); 2 Environmental Microbial and Food Safety Laboratory, Agricultural Research Service, USDA, Beltsville, MD 20705, USA; kevin.chao@ars.usda.gov (K.C.); jianwei.qin@ars.usda.gov (J.Q.); 3 National Institute of Agricultural Science, Rural Development Administration, 310 Nongsaengmyeong-ro, Wansan-gu, Jueonju-si, Jeollabuk-do 54875, Korea; cymoh100@korea.kr; 4 School of Biosystems and Food Engineering, University College Dublin, Dublin 4, Ireland; carlos.esquerre@ucd.ie; 5 Food Quality, Agricultural Research Service, USDA, Beltsville, MD 20705, USA; stephen.delwiche@ars.usda.gov

**Keywords:** penetration depth, hyperspectral imaging, milk powder, PLS-DA

## Abstract

The increasingly common application of the near-infrared (NIR) hyperspectral imaging technique to the analysis of food powders has led to the need for optical characterization of samples. This study was aimed at exploring the feasibility of quantifying penetration depth of NIR hyperspectral imaging light for milk powder. Hyperspectral NIR reflectance images were collected for eight different milk powder products that included five brands of non-fat milk powder and three brands of whole milk powder. For each milk powder, five different powder depths ranging from 1 mm–5 mm were prepared on the top of a base layer of melamine, to test spectral-based detection of the melamine through the milk. A relationship was established between the NIR reflectance spectra (937.5–1653.7 nm) and the penetration depth was investigated by means of the partial least squares-discriminant analysis (PLS-DA) technique to classify pixels as being milk-only or a mixture of milk and melamine. With increasing milk depth, classification model accuracy was gradually decreased. The results from the 1-mm, 2-mm and 3-mm models showed that the average classification accuracy of the validation set for milk-melamine samples was reduced from 99.86% down to 94.93% as the milk depth increased from 1 mm–3 mm. As the milk depth increased to 4 mm and 5 mm, model performance deteriorated further to accuracies as low as 81.83% and 58.26%, respectively. The results suggest that a 2-mm sample depth is recommended for the screening/evaluation of milk powders using an online NIR hyperspectral imaging system similar to that used in this study.

## 1. Introduction

Milk, both a nutritious food in itself and a functional ingredient in other food products, is a complex fluid consisting of fats, proteins, minerals, vitamins, enzymes, carbohydrates and water. However, fluid milk is difficult to transport and store. Therefore, milk powders are produced using drying technologies to turn fluid milk into dry milk powder. Nonfat milk and whole milk are the two most common milk powders and contribute nutritionally to many food formulations, including reconstituted milk, dairy products, baked goods, confectionery, processed meat products, nutritional beverages and prepared ready-to-eat foods. As an important food ingredient for human and animal food, milk powder safety is a worldwide concern. In recent years, incidents of milk powder adulteration by melamine (2,4,6-triamino-1,3,5-triazine) to boost apparent protein content caused illnesses and resulted in wide recognition of melamine contamination as a food safety problem. Traditional methods of melamine detection in foods involve analytical techniques, such as mass spectrometry and high performance liquid chromatography, are time consuming, expensive and require complicated sample preparation procedures [1,2]. Visible (VIS) and near-infrared (NIR) spectroscopy have been studied as non-destructive methods to detect melamine in milk by several research groups [3,4]; however, spectroscopic assessments with relatively small point-source measurements cannot provide information on the spatial distribution of melamine particles within a food sample.

As one of the most promising tools for non-destructive real-time evaluation of food quality and safety in numerous applications, hyperspectral imaging combines features of both imaging and spectroscopy, such that it is capable of not only directly assessing the presence of different components simultaneously, but also locating the spatial distribution of those components in the products under examination [5]. Hyperspectral imaging may be operated in either reflectance or transmittance modes, although reflectance is more commonly used. Generally, transmittance mode is used for thinly-prepared samples, allowing light to pass through the samples [6,7,8,9], while diffuse reflectance is used for thicker samples in hyperspectral imaging measurements of whole or larger portions of foods, such as apples [10], peach [11], mushrooms [12], cucumbers [13] and chickens [14]. Hyperspectral reflectance imaging has been used to detect defects and contaminants and to evaluate quality attributes for fruits, vegetables, meats and dairy products. Implemented with automated image processing and analysis algorithms, hyperspectral imaging has been demonstrated for effective real-time assessment of the quality and safety attributes of poultry [14]. For reflectance imaging, the NIR light must sufficiently penetrate the food material in order for the intensity of the remitted radiation, as a function of wavelength (or frequency), to have been influenced by the chemical nature of the absorbing compound.

Light penetration depth is defined as the depth in a sample material at which incident light is reduced by 99% and will vary with the status of sample, the type of sample and the detection wavelength [15]. Hyperspectral reflectance imaging is usually operated in the VIS-NIR (400–1000 nm) or NIR (1000–1700 nm) range. Limited research exists for the investigation of the penetration depth in the VIS and NIR ranges. Lammertyn *et al.* [16] reported light penetration depth in apples to be up to 4 mm in the 700–900-nm range and between 2 and 3 mm in the 900–1900-nm range. Qin and Lu [15] found that the light penetration depth in fruit tissue varies depending on the type of fruit, ranging from 7.1 mm for plums to 65.2 mm for zucchini. Most studies reporting light penetration depths were conducted on fruits. Further research would provide not only helpful references for thickness determination, but also valuable insight into appropriate sensing configurations, especially for milk powder products [17,18]. Fu *et al.* [18] coupled the NIR hyperspectral imaging technique (990–1700 nm) with spectral similarity analyses to detect melamine mixed into samples of dry milk powder. Imaging allowed visualization of the distribution of melamine particles in the milk mixture samples that were prepared at melamine concentrations ranging from 0.02%–1.00% and presented for imaging in plastic Petri dishes. However, it was not examined exactly how many millimeters the NIR hyperspectral imaging light could penetrate into the milk powder samples. The objective of this study was to determine the penetration depth of NIR hyperspectral imaging light in milk powder for the wavelengths between 937.5 nm and 1653.7 nm.

## 2. Experimental Section

### 2.1. Sample Preparation

**Figure 1:**
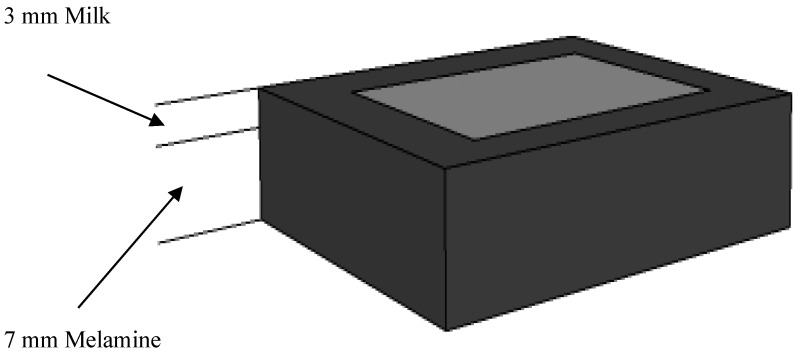
Milk-melamine sample holder (e.g., 3 mm-thick milk and 7 mm-thick melamine layers). The light grey area shows the sample surface (30 mm × 30 mm).

Eight different milk powder products were purchased from commercial retailers, including 5 brands of nonfat milk powders (‘valley (N)’, Organic Valley, La Farge, WI, USA; ‘nestle (N)’, Nestle, Solon, OH, USA; ‘hoosier (N)’, Hoosier Hill Farm, Fort Wayne, IN, USA; ‘now (N)’, Now Real Food, Blooming, IL, USA; ‘bob (N)’, Bob’s Red Mill, Pheasant Court Milwaukie, OR, USA) and three brands of whole milk powders (‘hoosier (W)’, Hoosier Hill Farm, Fort Wayne, IN, USA; ‘nestle (W)’, Nestle, Glendale, CA, USA; ‘peak (W)’, Peak, Friesland Campina, P. Stuyvesantwet 1, AC Leeuwarden, Holland). Melamine powder (99% purity) was obtained from Sigma-Aldrich Company (M2659, St. Louis, MO, USA). For detecting the light penetration depth of the hyperspectral imaging system, different thicknesses (1 mm, 2 mm, 3 mm, 4 mm and 5 mm) of pure milk powders were layered on the top of pure melamine. These milk-melamine samples were prepared in black electroplated aluminum plates precisely machined with square wells (30-mm width and height, 10-mm depth). Each sample was first prepared with a leveled layer of pure melamine in the bottom of the well, followed by a leveled layer of pure milk powder on top of the melamine. The combined thicknesses of the melamine and milk powder layers completely filled the 10-mm depth of each well, with the milk depth ranging between 1 and 5 mm (*i.e*., 1 mm-thick milk layer over 9 mm of melamine, 2 mm-thick milk layer over 8 mm of melamine, and so on). Figure 1 illustrates the preparation of a sample containing 3 mm of milk over 7 mm of melamine. For each kind of milk powder, three samples were prepared at each of the five milk depths, as well as three samples of pure dry milk (10 mm of milk with no melamine underneath) and one sample of pure melamine. With 19 plates prepared for each of the milk powder products, a total of 152 samples were measured for the eight milk products used in this study.

### 2.2. Instrument and Experiment

**Figure 2:**
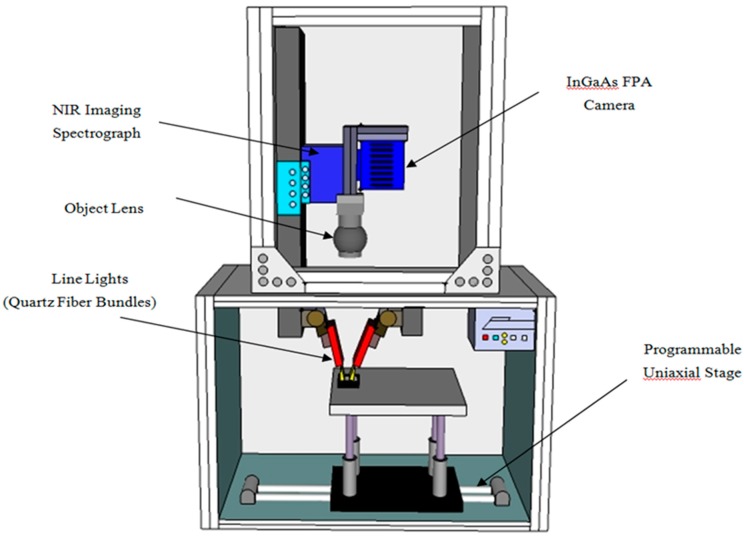
Schematic of the NIR hyperspectral imaging system used to acquire reflectance images of milk powders. FPA, focal-plane-array.

As shown in the schematic in Figure 2 and described in detail in Kim *et al.* [19], the line-scan NIR hyperspectral imaging system consists of an InGaAs focal-plane-array (FPA) camera with 320 × 256 pixels (Xenics, Model Xeva-1.7-320, Leuven, Belgium), an imaging spectrograph (SWIR Hyperspec, Headwall Photonics, MA, USA) and a 25-mm zoom lens (Optec, Model OB-SWIR25/2, Parabiago, Italy), as well as a computer for controlling the camera and acquiring images, two 150-W DC light sources with fiber optic bundles (Dolan Jenner, Model DC-950, Boxborough, MA, USA) and a motorized uniaxial stage (Velmex, Model XN10-0180-M02-21, Bloomfield, NY, USA). The camera array sensor consists of 150 usable pixels in the spectral dimension over a wavelength range of 937.5–1653.7 nm, with an average wavelength spacing of 4.8 nm. Except for the two 150-W quartz tungsten halogen light sources, the system is entirely housed with an aluminum-framed enclosure. The light is conveyed via the low-OH fiber optic bundles, with each bundle terminating in a 250 mm-long line of fibers encased in a machined aluminum head. The angle of incidence from the line lights is 30° (from surface normal), and the distance between line lights and sample surface is 20 mm. For each light source, a mechanical iris was used to allow approximately 75% of 150-W light intensity to arrive at the sample surface. The motorized stage moved the samples incrementally across the linear field of view for step-by-step acquisition of line-scan images.

In this study, square imaging pixels were achieved by setting the incremental step size to 0.1 mm to match the pixel-to-pixel distance along the imaging line, which included 320 pixels. The camera exposure time was set at 2.5 ms, and a total of 360 scans were acquired in 1 min. The camera digitized raw energy readings in 14-bit resolution. Finally, each sample’s spectral data were stored as a 16-bit hyperspectral image cube of dimensions 320 × 360 × 150 containing spatial and spectral data. The hypercube is a three-dimensional image, which represents a 2D spatial image with x-axis and y-axis coordinate information and z-axis spectral information.

### 2.3. Hyperspectral Data Analysis

#### 2.3.1. Image Preprocessing

In this study, dark current and white reference images were collected to correct the raw reflectance images for wavelength-dependent system responses and heterogeneous dark current in the FPA camera. The dark current image was captured while the lens was covered by a lens cap. An image of an illuminated 99% diffuse reflectance standard (Spectralon^TM^, SRT-99-120, Labsphere, NH, USA) was acquired for use as the white reference image. These images were used to calculate the relative reflectance image of each sample, which was calculated by dividing the difference in energy readings between sample and dark current by the difference in energy readings between the white reference and dark current.

#### 2.3.2. Spectral Preprocessing

The region of interest (ROI) for each sample was composed of a square region of 10,000 pixels (100 ×100 pixels around the image center), selected within the 30 mm × 30 mm sample area (shown in Figure 1) so as to include only milk powder areas and exclude background (plate) regions for further analyses. Spectral preprocessing techniques were used prior to develop a calibration model in the quest for improving the subsequent classification model. Common preprocessing methods for NIR spectra were used, including Standard Normal Variate (SNV), Multiplicative Scatter Correction (MSC), Extended MSC (EMSC), Normalized (NORMALIZED), the common (base 10) Logarithm (LOG10), Savitzky–Golay Smooth (SMOOTH) and the Savitzky–Golay 1st (1ST) and the Savitzky–Golay 2nd (2ND) derivative [20].

#### 2.3.3. Development of the Classification Model

After spectral preprocessing, partial least squares discriminant analysis (PLS-DA) was used to classify each pixel as belonging to either the pure milk class or the milk-melamine mixture class. PLS-DA, an extension of PLS modeling, aims to find the variables and directions in a multivariate space that discriminate the known classes in the calibration set. PLS components are computed under the constraint of the maximization of covariance between inputs and outputs. Therefore, it can provide a set of orthogonal factors that have the best predictive power from the combinations of different methods with an increased number of variables [21,22,23]. 

Prior to model development, the triplicate samples (three samples imaged at each depth of milk over melamine and three samples for each pure milk powder product) were divided into two groups, with two samples (comprising 20,000 ROI pixels) assigned to a model development dataset and one sample (comprising 10,000 ROI pixels) assigned to a validation dataset for use in evaluating model performance. Model performance was compared based on classification accuracies, *i.e.*, the percentage of correctly-classified pixels over the total number of pixels. To better assess the performance of the classification models, calibrations and validations for each depth were run three times [24]. For each of the 5 different milk-melamine preparations, a separate PLS-DA model (1-mm model, 2-mm model, 3-mm model, 4-mm model and 5-mm model) was developed. The number of components chosen for the PLS-DA models was determined by a contiguous block cross-validation method, in which each block contained the samples from one milk powder product. 

Image processing, selection of the square ROI, the spectral preprocessing operation and model development were performed in MATLAB (R2007b, MathWorks, Natick, MA, USA) equipped with the PLS Toolbox (v. 7.5, Eigenvector Research, Manson, WA, USA). 

## 3. Results and Discussion

### 3.1. Hyperspectral Spectra

**Figure 3:**
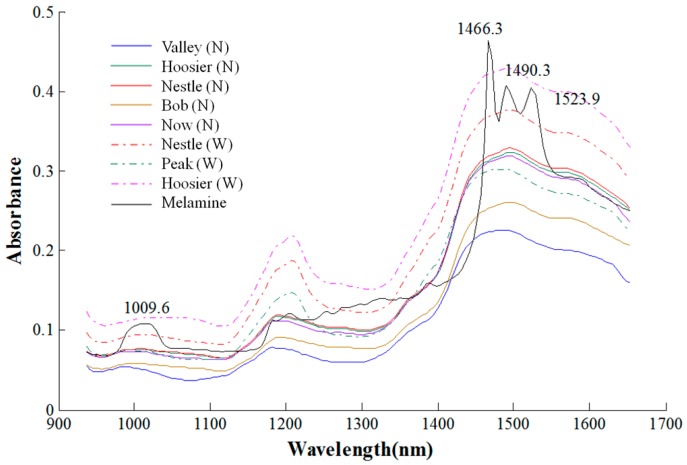
Representative mean spectra of pure nonfat (N) and whole (W) milk powders and pure melamine, each calculated from a 10,000-pixel ROI.

Figure 3 shows representative mean absorption spectra (calculated from 10,000-pixel ROI) of pure melamine and the eight pure milk products (including five nonfat and three whole) obtained from the LOG10 preprocessing technique. Significant differences, absorption peaks related to the first and second N-H functional group, can be clearly observed between melamine and milk spectra. The mean melamine spectrum had peaks near 1523.9 and 1490.3 nm, corresponding to the first overtone of N-H symmetric and anti-symmetric stretching vibration, respectively. The second overtone of N-H stretching vibration is located near 985.6–1033.7 nm (centered at 1009.6 nm). The most significant spectral difference between melamine and milk occurred near 1466.3 nm, which is attributed to aromatic amine structures [3] and showed the highest absorption in the melamine spectrum. For the nonfat milk spectra, the spectral patterns of the five different brands are similar, and another similar spectral pattern was observed for the three brands of whole milk from Figure 3. The absorbance of most nonfat milk spectra is lower than that of the whole milk spectra. For the visual difference between the nonfat and whole milk pattern, whole milk spectra have an evident absorption peak around 1211.5 nm, which is due to the second overtone of C-H stretching vibration constituted by saturated fat structures [25].

**Figure 4:**
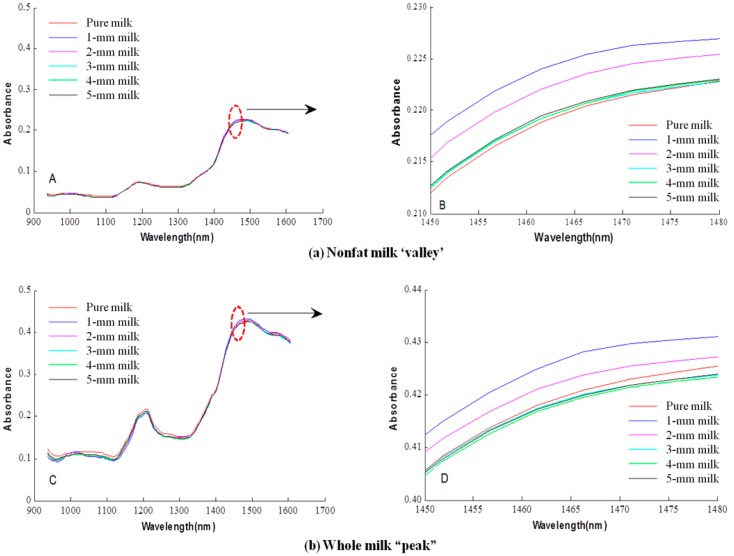
Mean ROI spectra of samples prepared using (**a**) ‘valley’ (N) nonfat milk and (**b**) ‘peak’ (W) whole milk, including pure milk samples and milk-melamine samples with milk depths from 1–5 mm (thickness). Plots A and C show the full spectra, while Plots B and D show the enlarged view of the mean spectra near the 1466.3-nm melamine peak.

Figure 4 shows representative mean spectra for one nonfat milk and one whole milk, valley (nonfat (N)) and peak (whole (W)), including milk-melamine samples for milk depths of 1–5 mm (thickness) and for pure milk. Among the eight milk products used in this study, ‘peak’ (W) had the strongest absorbance and ‘valley’ (N) had the weakest absorbance. The mean spectra of milk-melamine samples are very similar to the mean spectrum of pure milk; all exhibit no obvious melamine absorption features. This observation suggests that individual pixel-based spectral evaluations (instead of averaged spectra across spatial image areas) may allow better detection of melamine to measure the penetration depth of milk [18]. In the region of the 1466.3-nm melamine absorption peak, the mean absorbance notably decreased as the milk depth increased from 1 mm–3 mm. For 3 mm–5 mm, the mean spectra were nearly the same as that for pure milk. The same trends were observed for both nonfat milk ‘valley’ and whole milk ‘peak’.

### 3.2. Discriminant Models for Milk Depth Classification

**Figure 5:**
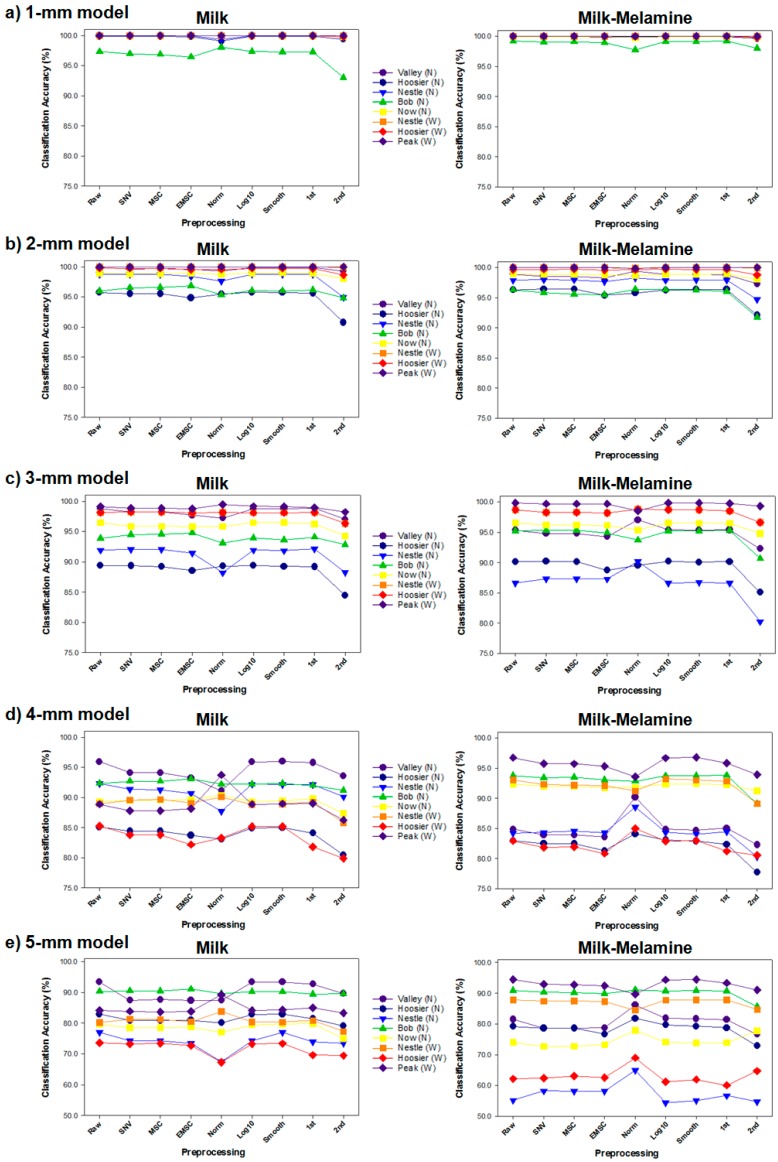
Classification comparison of classification results for PLS-DA models coupled with specific spectral preprocessing algorithms for milk-melamine samples at milk depths from 1 mm to 5 mm (**a**–**e**).

The plots in Figure 5 compare the validation set classification results (average of three runs) for the PLS-DA models coupled with specific spectral preprocessing algorithms, for milk-melamine at each of the five different milk depths (1-mm–5-mm models). As shown in Figure 5, the different preprocessing algorithms can have a great impact on model accuracy. Compared to the other spectra preprocessing algorithms, the 2ND derivative consistently resulted in low classification accuracy. The reason may be that it has a more prominent spectra shoulder after derivative transformation, but it affects the component number selection of PLS-DA. The models coupled with the SNV and MSC algorithm gave better, more robust performance than the NORMALIZED model (mean zero, unit variance), which is due to reducing the scattering influence from particle size.

Since the classification accuracies of the calibration set were better than those of the validation sets, only the classification results of the validation set for milk and milk-melamine samples are presented. Table 1 shows the classification results of the validation set based on the PLS-DA model coupled with the SNV preprocessing algorithm for the eight milk powders. The data show the classification accuracy notably decreasing as the milk depth increases from 1 mm–5 mm. For the 1-mm model, 99.65%–100% of milk samples (pixels) and 99.06%–100.00% of milk-melamine samples were correctly classified, for overall accuracies of 99.93% and 99.86% for milk and milk-melamine samples, respectively, across the eight milk powders. The 2-mm model’s highest accuracy was the same as that for the 1-mm model, while its lowest accuracies were lower at 95.53% for milk and 96.4% for milk-melamine samples. The average misclassification rates were 1.39% for milk and 1.58% for milk-melamine samples across all eight milk powders. Although the 3-mm model achieved an average accuracy 95.54% for milk and 94.93% for milk-melamine samples, the classification accuracies of nonfat milk ‘hoosier’ and ‘nestle’ were lower than 90%, which is not suitable for melamine detection at lower concentrations. As the milk depth increased to 4 mm and 5 mm, the nearly identical spectra for milk and for milk-melamine (shown in Figure 3) resulted in deteriorated model performance. The average misclassification rate of the 4-mm model was greater than 10%, while about 20% of the samples were misclassified by the 5-mm model for some of the milk powders. This means that the 4-mm and 5-mm models were invalid for classification.

**Figure 6:**
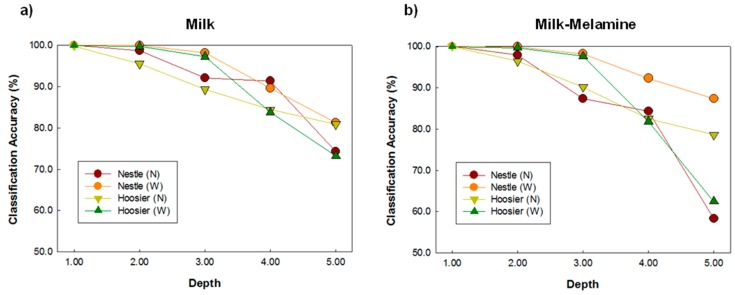
Comparison of the classification results for the same brand nonfat and whole milk using the PLS-DA model coupled with the SNV spectral preprocessing algorithm for (**a**) milk and (**b**) milk-melamine.

Figure 6 shows the classification results for two brands of nonfat and whole milk using the PLS-DA model coupled with the SNV spectra preprocessing algorithm. For the same brand milk powder, the classification results of whole milk were slightly higher than those of nonfat milk for the 1-mm–3-mm valid models.

## 4. Conclusions

In this study, the penetration depth of near-infrared hyperspectral imaging light (937.5–1653.7 nm) was investigated for milk powders. Five different depths of milk powder, from 1 mm–5 mm, were investigated for the detection of melamine underneath the milk powder. Classification models were developed using the PLS-DA technique for milk and milk-melamine samples prepared using five brands of non-fat milk powder and three brands of whole milk powder. The classification results showed that the classification accuracy gradually decreased as the milk depth increased. For the 2-mm models, the classification accuracies were higher than 95% for both milk and milk-melamine samples for all of the milk powders under investigation. It can be concluded that the use of a 2-mm milk powder depth can be recommended for applying NIR hyperspectral imaging for the detection of contaminants in milk powder. In addition, the method described can also be potentially applied to other food powders for penetration depth measurement of NIR hyperspectral imaging system.

## Figures and Tables

**Table 1:** Classification results of the validation set for milk and milk-melamine samples from a 1-mm–5-mm depth using PLS-DA coupled with the Standard Normal Variate (SNV) spectra preprocessing algorithm.

Classification (%)						
	Depth	1 mm	2 mm	3 mm	4 mm	5 mm
	valley (N)	99.98	99.66	98.20	94.13	87.41
	hoosier (N)	99.86	95.53	89.37	84.43	80.91
	nestle (N)	100.00	98.72	92.08	91.39	74.26
	bob (N)	99.65	96.51	94.49	92.70	90.52
Milk	now (N)	99.97	98.80	95.86	89.56	78.50
	nestle (W)	100.00	99.98	98.19	89.57	81.29
	hoosier (W)	99.98	99.73	97.29	83.77	73.19
	peak (W)	99.99	99.98	98.86	87.81	83.85
	Average	99.93	98.61	95.54	89.17	81.24
	valley (N)	99.91	98.56	94.81	83.94	78.60
	hoosier (N)	99.96	96.43	90.21	82.47	78.62
	nestle (N)	100.00	97.97	87.34	84.35	58.26
Milk-	bob (N)	99.06	95.82	95.29	93.42	90.30
melamine	now (N)	99.94	98.89	96.21	92.03	72.73
	nestle (W)	100.00	100.00	98.27	92.32	87.42
	hoosier (W)	99.98	99.70	97.66	81.83	62.43
	peak (W)	99.99	99.99	99.70	95.76	92.92
	Average	99.86	98.42	94.93	88.26	77.66
